# Supplementary material for: Glioblastoma Subtypes Exhibit Distinct Migration Mechanics and Immune Responses
Source: Cancer Res Commun. 2026 Aug 11;6(8):1898–915. doi: 10.1158/2767-9764.CRC-25-0579 (PMC13489672; doi:10.1158/2767-9764.CRC-25-0579)
Supplement: Supplementary file 1 [file crc-25-0579_table_s1_suppst1.docx]

**Table S1:** IHC antibodies and reagents used in Figure 1A and Figure 7D

| IHC | Antibody | Antigen retrieval | RRID | Blocking | Detection |
| --- | --- | --- | --- | --- | --- |
| Ki67 | CRM325 (Biocare) | High pH EDTA | AB_2721189 | Dako protein block | Dako rabbit envision |
| CD3 | A0452 (Dako) | High pH EDTA | AB_2335677 | Dako protein block | Biocare rat detection |
| IBA-1 | AB107159 (Abcam) | Low pH citrate | AB_10972670 | Dako protein block | Biocare goat detection |
| Cleaved Caspase-3 (Asp175) | #9661 (Cell Signaling Technology) | High pH EDTA | AB_2341188 | Dako protein block | Dako rabbit envision |
| Granzyme B | D6E9W (Cell Signaling Technology) | Low pH citrate | AB_2799313 | Vectastain, goat serum | Vectastain ABC-HRP |
