# Supplementary material for: Glioblastoma Subtypes Exhibit Distinct Migration Mechanics and Immune Responses
Source: Cancer Res Commun. 2026 Aug 11;6(8):1898–915. doi: 10.1158/2767-9764.CRC-25-0579 (PMC13489672; doi:10.1158/2767-9764.CRC-25-0579)
Supplement: Supplementary file 2 [file crc-25-0579_table_s5_suppst5.docx]

**Table S5:** Characteristics of patient-derived xenograft (PDX) lines used in this study

| GBM | Xenograft start | Sex | Molecular subtype  (RNAseq) |
| --- | --- | --- | --- |
| GBM16 | 2002 | F | mesenchymal |
| GBM39 | 2003 | M | mesenchymal |
| GBM44 | 2003 | F | mesenchymal |
| GBM64 | 2006 | F | proneural |
| GBM80 | 2007 | M | proneural |
| GBM85 | 2008 | M | proneural |
