# Supplementary material for: Glioblastoma Subtypes Exhibit Distinct Migration Mechanics and Immune Responses
Source: Cancer Res Commun. 2026 Aug 11;6(8):1898–915. doi: 10.1158/2767-9764.CRC-25-0579 (PMC13489672; doi:10.1158/2767-9764.CRC-25-0579)
Supplement: Supplementary file 3 [file crc-25-0579_table_s6_suppst6.docx]

**Table S6:** Cell migration simulator parameter values

| Symbol | Parameter | Value |
| --- | --- | --- |
| *N_m_* | Total number of motors | 10,000 |
| *N_c_* | Total number of clutches | 250; 750 |
| *A_tot_* | Total possible actin protrusion length | 100 μm |
| *v_p_** | Maximum actin polymerization velocity | 200 nm/s |
| *k_mod_** | Maximum module birth rate | 1 s^-1^ |
| *k_cap_* | Module capping rate | 0.001 s^-1^ |
| *l_in_* | Initial module length | 5 μm |
| *l_min_* | Minimum module length | 0.1 μm |
| *κ_cell_* | Cell spring constant | 10,000 pN/nm |
| *n_c,cell_* | Number of cell body clutches | 10; |
| *n_m_** | Maximum number of module motors | 0.1**N_c_* |
| *F_m_* | Motor stall force | 2 pN |
| *v_m_** | Unloaded motor velocity | 120 nm/s |
| *n_c_** | Maximum number of module clutches | 75; 750 |
| *k_on_* | Clutch on-rate | 1 s^-1^ |
| *k_off_** | Clutch unloaded off-rate | 0.1 s^-1^ |
| *κ_c_* | Clutch spring constant | 0.8 pN/nm |
| *F_b_* | Characteristic clutch rupture force | 2 pN |
| *κ_s_* | Substrate spring constant | Variable |
