# Supplementary material for: Glioblastoma Subtypes Exhibit Distinct Migration Mechanics and Immune Responses
Source: Cancer Res Commun. 2026 Aug 11;6(8):1898–915. doi: 10.1158/2767-9764.CRC-25-0579 (PMC13489672; doi:10.1158/2767-9764.CRC-25-0579)
Supplement: Supplementary file 4 [file crc-25-0579_table_s7_suppst7.docx]

**Table S7:** Brownian dynamics tumor simulator parameters values

| **Parameter Name** | **Parameter** | **Value** | **Units** | **Reference** |
| --- | --- | --- | --- | --- |
| Cancer cell radius | r_cancer_ | 10 | µm |  |
| T cell radius | r_CTL_ | 3 | µm |  |
| Cancer cell motility | D_cancer_ | 7.8; 42 | µm^2^/h | This study |
| T cell motility | D_CTL_ | 300 | µm^2^/hr | (Boissonnas *et al.*, 2007; Halle *et al.*, 2016) |
| Cancer cell proliferation | p_cancer_ | 0.5 | 1/d | This study |
| T cell proliferation | p_CTL_ | 1 | 1/d | (Riggs *et al.*, 2008) |
| Contact duration | 1/k_dissoc_ | 10 | min | (Halle *et al.*, 2016; Halle, Halle and Förster, 2017) |
| Cancer cell HP* | HP_cancer_ | 1 | contacts | (Halle *et al.*, 2016) |
| T cell HP* | HP_CTL_ | 20 | contacts | Unknown, adjustable |
